# Separation and Characterization of Angiotensin I Converting Enzyme (ACE) Inhibitory Peptides from *Saurida elongata* Proteins Hydrolysate by IMAC-Ni^2+^

**DOI:** 10.3390/md15020029

**Published:** 2017-02-15

**Authors:** Lixia Sun, Shanguang Wu, Liqin Zhou, Feng Wang, Xiongdiao Lan, Jianhua Sun, Zhangfa Tong, Dankui Liao

**Affiliations:** 1 Guangxi Colleges and Universities Key Laboratory of New Technology and Application in Resource Chemical Engineering, School of Chemistry and Chemical Engineering, Guangxi University, Nanning 530004, China; binglin0628@163.com (L.S.); zhouliqin100430@163.com (L.Z.); lanxiongdiao@163.com (X.L.); sunjhgx@163.com (J.S.); zhftong@sina.com (Z.T.); 2 Medical College, Guangxi University of Science and Technology, Liuzhou 545006, China; 3 College of Materials Science and Engineering, Beijing University of Chemical Technology, Beijing 100029, China; wangf@mail.buct.edu.cn

**Keywords:** ACE-inhibitory activity, lizard fish, hydrolysate, purification, IMAC

## Abstract

Lizard fish protein hydrolysates (LFPH) were prepared from Lizard fish (*Saurida elongata*) proteins possessing powerful angiotensin I converting enzyme (ACE) inhibitory activity and the fraction (LFPH-I) with high ACE inhibitory activity was obtained through ultrafiltration. The active Fraction (F2) was isolated from LFPH-I using immobilized metal affinity chromatography (IMAC**-**Ni^2+^). Analysis of amino acid levels revealed that F2 eluted from IMAC was enriched in Met, His, Tyr, Pro, Ile, and Leu compared to the crude peptide LFPH-I. F2 with the high ACE inhibitory activity (IC_50_ of 0.116 mg·mL^−1^) was further separated by a reverse-phase column to yield a novel ACE inhibitory peptide with IC_50_ value of 52 μM. The ACE inhibitory peptide was identified as Arg-Tyr-Arg-Pro, RYRP. The present study demonstrated that IMAC may be a useful tool for the separation of ACE inhibitory peptides from protein hydrolysate.

## 1. Introduction

Bioactive peptides are described as “food-derived components” or “nutrition for optimal health” due to their nutritional value, exerting a physiological effect in the body and making a further contribution to disease prevention [1]. Bioactive peptides can be released from primary or parent proteins by hydrolysis, and a number of them have been characterized in natural and modified food in recent years.

Angiotensin-I-converting enzyme inhibitors—which can reduce the activity of the angiotensin-I-converting enzyme (ACE)—have an antihypertensive effect in vivo, and play a key role in the treatment of the hypertension [2]. ACE inhibitory peptides—one of the most important ACE-inhibitors—can be derived from enzymatic hydrolysates of food protein, such as soybean, fish, snakes, milk, and vegetable protein [3,4].

Improving the separation efficiency is the bottleneck in the separation of bioactive peptides from hydrolysates. Some methods have been widely used, such as membrane separation technology [5], gel chromatography [6], ion exchange chromatography [7,8], high performance liquid chromatography [9], and capillary electrophoresis [10]. However, sometimes a combination of these methods and several purification steps are necessary to obtain pure peptides. The separation process would be time-consuming and high-costly. 

It is possible to rapidly separate pure peptides from protein hydrolysates for the development of affinity separation technology. In the mid-1970s, Porath et al. first described immobilized metal ion affinity chromatography (IMAC) [11]. Since that time, proteins of interest have been enriched through IMAC [12,13,14,15,16]. Nickel ion immobilized on solid supports have been widely used in IMAC [17,18]. Recently, some research about the separation and purification of peptides by IMAC has been reported [19]. Furthermore, IMAC had been used to fractionate ACE inhibitory peptides from hydrolysates of protein [20,21].

Based on the separation of the ACE inhibitory peptides which is a previous work of our project group [22], IMAC was used to isolate ACE inhibitory peptides from lizard fish hydrolysates in the present study. There are several advantages to using IMAC, including efficiency, high purity, and low cost.

## 2. Results and Discussion

### 2.1. Purification of ACE Inhibitory Peptides by Immobilized Ni^+^ Ions on IDA-Conjugated Agarose Microspheres (AS-IDA-Ni^2+^)

**Figure 1:**
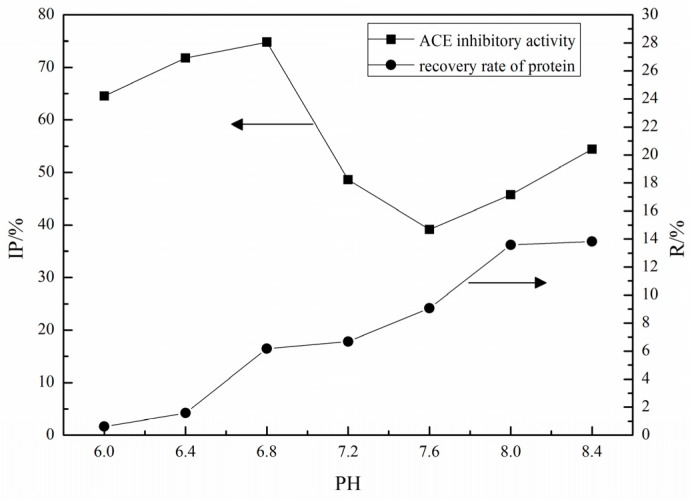
Effects of pH values on recovery rate of protein and angiotensin-I-converting enzyme (ACE) IP of elution fractions.

By using 0.02 M phosphate-buffered saline (PBS) (pH 4.0, 0.5 M NaCl) as elution buffer, the effect of pH on the binding capacity of lizard fish protein hydrolysates (LFPH) was shown (Figure 1); the recovery rate (R) of protein enriched by affinity column increased with the increase of pH. However, the elution fractions with high recovery rate would not result in high ACE inhibitory activity (IP). ACE inhibitory activity of the elution fraction reached the highest level at pH 6.8, and equilibration buffers with pH 6.8 was chosen for further study.

**Figure 2:**
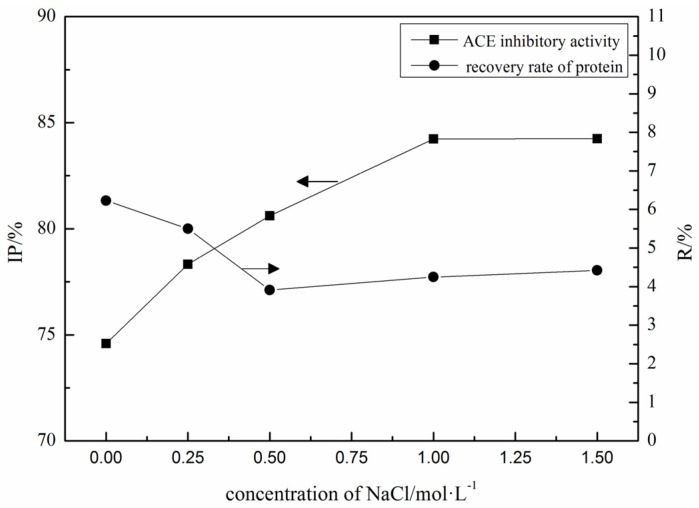
Effects of NaCl concentration in equilibrating buffer on the recovery rate of protein and ACE inhibitory activity of elution fractions.

By using 0.02 M PBS (pH 4.0, 0.5 M NaCl) as elution buffer, the ACE inhibitory activity of elution fractions increased with the increase of salt (NaCl) concentration, and reached the highest level at 1 M of NaCl in equilibration buffers (Figure 2). However, the recovery rate of protein did not change significantly over the concentration of NaCl. The equilibration buffers with 1 M NaCl was chosen. 

**Figure 3:**
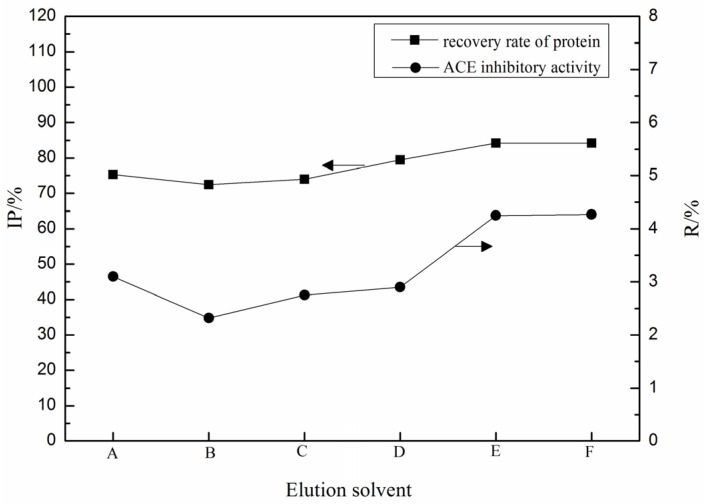
Effects of eluent on the recovery rate of protein and ACE inhibitory activity of immobilized metal affinity chromatography (IMAC) fractions by using phosphate-buffered saline (PBS) buffer (pH 6.8, 0.5 M NaCl) as equilibration buffer: (A) iminazole; (B) 0.02 mol·L^−1^ sodium phosphate buffer at pH 6.0; (C) 0.02 mol·L^−1^ sodium phosphate buffer at pH 5.0; (D) 0.02 mol·L^−1^ sodium phosphate buffer at pH 4.0; (E) 0.02 mol·L^−1^ sodium phosphate buffer at pH 4.0, containing 0.50 mol·L^−1^ NaCl; and (F) 0.02 mol·L^−1^ sodium phosphate buffer at pH 4.0, containing 1.0 mol·L^−1^ NaCl.

By using PBS buffers (pH 6.8, containing 0.5 M NaCl) as equilibration buffers, the effect of elution buffers on the elution of the inhibitory fraction of LFPH (LFPH-I) was studied with imidazole or 0.02 M PBS containing NaCl, the concentration from 0 to 1.0 M. The peptides eluted by imidazole or 0.02 M phosphate buffers (pH ≤ 6.0) (Figure 3). With the increase of salt (NaCl) concentration of elution buffers and decrease of pH value, the recovery rate of protein increased and reached the highest level at 0.5 M NaCl. Peptides showed the highest inhibitory activity when eluted by 0.02 M phosphate buffers (pH 4.0) containing 0.5 M NaCl.

**Figure 4:**
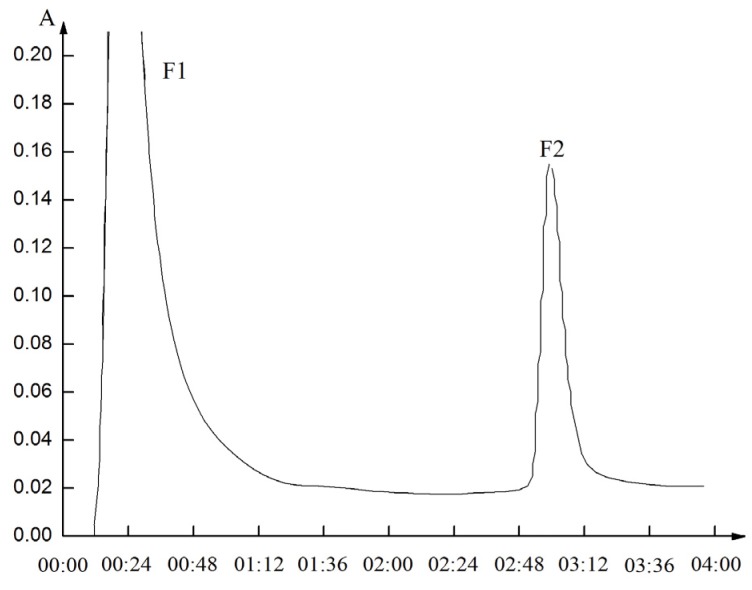
IMAC Chromatogram of the inhibitory fraction of lizard fish protein hydrolysate (LFPH-I) using PBS buffer (pH 6.8, 1 M NaCl) as equilibration buffer and 0.02 M PBS (pH 4.0, 0.5 M NaCl) as elution buffer.

According to the above results, 0.02 M phosphate buffer at pH 6.8, containing 1 M NaCl was selected as the equilibrating buffer, and 0.02 M phosphate buffer at pH 4.0, containing 0.5 M NaCl was selected as the elution buffer. Under this condition, LFPH-I was fractionated to two peaks (fraction 1 and fraction 2) (Figure 4). The crude protein LFPH-I and eluted peptides (Fraction 2) with IC_50_ of 0.123 mg∙mL^−1^ and 0.116 mg∙mL^−1^, respectively. The IC_50_ values of the eluted peptides F2 (Fraction 2) obtained after the separation of LFPH-I by AS-IDA-Ni^2+^ had 13.82% lower than crude protein LFPH-I. The activity of enrichment of components increased, which indicates that AS-IDA-Ni^2+^ can enrich active components.

### 2.2. Amino Acid (AA) Composition

F2 was analyzed for AA composition. AA analysis revealed that peptide F2 eluted from IMAC was enriched in Met, His, Tyr, Pro, Ile, and Leu compared to the crude peptide LFPH-I (Table 1). An IMAC fraction which had 16 times more ACE inhibitory activity than the parent protein hydrolysate was separated from the enzymatic hydrolysates of wheat gliadin hydrolysates by immobilized Ni^2+^-ion affinity chromatography, and it was found that this fraction is enriched in histidine and hydrophobic AA (Pro, Val, Ile, Leu, and Phe) [11]. The results of this study have a slight difference from the findings reported by Thewissen [11]. The major reasons were the difference of interaction system and adsorption conditions of AS-IDA-Ni^2+^.

### 2.3. Separation of Metal Chelating Peptides (F2) by Reverse Phase (RP)-HPLC

**Figure 5:**
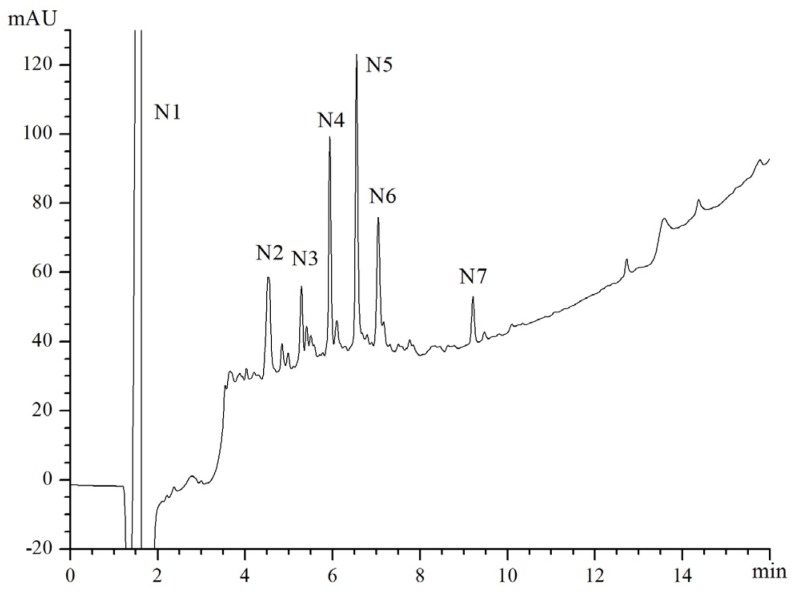
The reverse phase (RP)-HPLC chromatogram of the fraction separated by IMAC.

F2 was further separated by RP-HPLC on a Zorbax SB C18 column. The RP-HPLC profile of the fractions collected from F2 was shown (Figure 5). Among those fractions separated by RP-HPLC, fraction N5 showed the highest ACE inhibitory activity, with IC_50_ value of 0.031 mg∙mL^−1^ (52 μM) (Table 2), and was collected for amino acid sequence analysis.

### 2.4. Amino Acid Sequence Analysis

**Figure 6:**
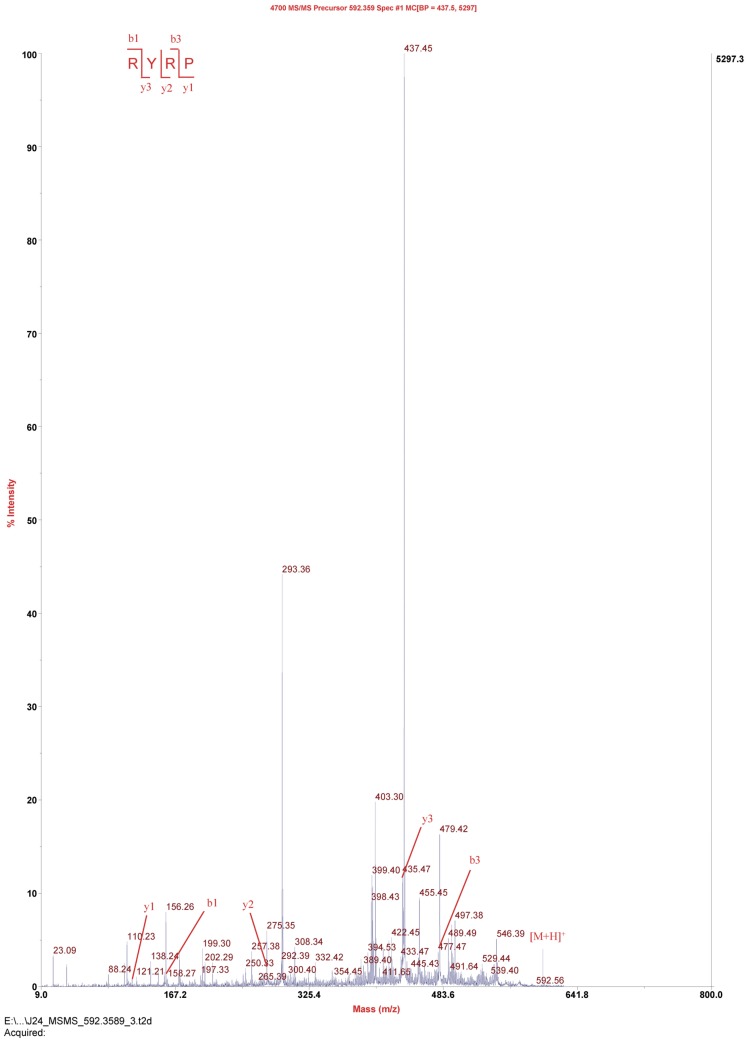
Peptide profile of peak N5 performed by MALDI TOF/TOF mass spectrometry analysis.

The amino acid sequences of the active fraction N5 were identified as Arg-Tyr-Arg-Pro (RYRP). The molecular mass (592.0 Da) corresponded to its sequence (Figure 6). The quantitative structure–activity relationship (QSAR) of ACE inhibitory peptides indicates that ACE inhibition of the peptides relates to their amino acid sequences—the *C*-terminal region of the peptide plays a predominant role in binding to the enzyme. The research suggested that hydrophobic amino acids or aromatic amino acids in the *C*-terminal will be preferred for increasing the peptide activity. On the other hand, *C*-terminal with positively charged amino acids such as lysine (K) and arginine (R) may contribute to an increase in the ACE inhibitory potential. In the current study, RYRP is composed of four amino acid residues with hydrophobic residue, proline as the *C*-terminal residue, exhibiting strong ACE-inhibitory activity (IC_50_ of 52 μM). Its ACE inhibitory activity was stronger than RVCLP [23], but weaker than SPRCR [22]. This phenomenon is in accordance with the QSAR studies. Meanwhile, the IC_50_ value of RYRP falls within 0.3 to 1500 μM, which is the range of IC_50_ values of ACE inhibitor peptides from marine proteins [24].

## 3. Materials and Methods

### 3.1. Materials

Lizard fish was purchased from a local market in Nanning, China, during the fishing season. It was identified by engineer Cuican at Beihai Institute of Oceanology. Its muscle was rapidly separated, dried with hot air (90 °C) over 8 h, powdered, and then stored in the cryogenic refrigerator. Agarose (BR) and NiSO_4_·6H_2_O (AR) were purchased from Sinopharm Chemical Reagent Co., (Beijing, China). Iminodiacetic acid (IDA) was purchased from Sinopharm Chemical Reagent Co., Ltd. (Beijing, China). ACE (from rabbit lung; 2.0 units/mg of protein) and hippuryl-l-histidyl-l-leucine (HHL) were purchased from the Sigma Chemical Company (St. Louis, MO, USA). All other reagents used were of analytical grade.

### 3.2. Preparation of Agarose-Based IMAC Adsorbents

Agarose microspheres (AS) were prepared by an emulsification procedure according to Ying et al. [25]. Briefly, 10 mL of agarose solution (2.5%, *w*/*v*) was prepared by heating agarose solution in water to 95–100 °C. The solution was then transferred to a mixture of 50 mL of liquid paraffin and 0.75 g Span-80 in a three-necked flask. Under 400 r·min^−1^ stirring, the mixture was emulsified for 30 min and then immediately cooled to 4 °C. The AS were isolated by filtration and washed with petroleum ether, isopropanol, and then with deionized water (DI water).

AS were activated by suspending microspheres in a mixture of 80 mL 0.8 M NaOH solution, 20 mL epichlorohydrin, and 0.3 g NaBH_4_. The reaction was allowed to proceed at 40 °C for 4 h. The activated microspheres, collected by filtration, were rinsed with DI water to remove the excessive epichlorohydrin. The activated microspheres were suspended in 200 mL 2 M sodium carbonate solution containing 10.0 g iminodiacetic acid (IDA) and were allowed to react at room temperature for 12 h under magnetic stirring.

The IDA-conjugated agarose microspheres (AS-IDA) were then recovered by filtration and rinsed sequentially with water. The immobilization of metal ions Ni^2+^ on AS-IDA (AS-IDA-Ni^2+^) was carried out by submerging AS-IDA in 250 mL of 0.02 mol·L^−1^ NiSO_4_ at room temperature for 12 h. AS-IDA-Ni^2+^ was recovered by filtration and rinsed sequentially with DI water.

The amount of Ni (II) immobilized on AS-IDA-Ni^2+^ was determined as follows: 2.0 g of the resulting resin (AS-IDA-Ni^2+^) was mixed with 10 mL of 0.05 mol·L^−1^ EDTA. The mixture was shaken in a thermostated shaker (120 rpm) at 30 °C for 1 h. The concentration of Ni (II) in the filtrate was analyzed using UV spectrophotometry at 594 nm.

### 3.3. Preparation of Hydrolysates from Lizard Fish Muscle Protein

Preparation of Hydrolysates from lizard fish muscle protein was previously described by Wu et al. [22]. Briefly, Lizard fish (*Saurida elongata*) muscle protein was hydrolyzed using neutral protease under the following conditions: enzyme-to-substrate ratio of 10,000 U·g^−1^, 48 °C, pH 7.0, and hydrolysis time of 2 h. Hydrolysates were fractionated by ultrafiltration, and the fraction (LFPH-1) which was able to pass through the 5 kDa membrane was used for further IMAC separation.

### 3.4. IMAC Chromatographic Procedures

An affinity column was prepared by filling AS-IDA-Ni^2+^ in a glass chromatography column (1.6 × 20 cm, Shanghai Troody Analytical Instrument Co., Ltd, Shanghai, China) and connected to MB 99-1 automatic separation of nucleic acid protein chromatography (Shanghai Huxi Analysis Instrument Factory Co., Ltd., Shanghai, China) equipped with UV detector (280 nm) and fraction collector.

The affinity column was equilibrated at a flow rate of 1.5 mL·min^−1^ with PBS. LFPH-I were suspended in 1.0 mL of the same buffer and loaded on the column. In the next step, the affinity column was rinsed with the buffer (1.5 mL·min^−1^) to remove unbound material. Fractions with affinity for Ni^2+^ ion were eluted and monitored at 280 nm. The fractions collected from the IMAC-Ni^2+^ were pooled and freeze-dried. The ACE-inhibitory activity of these fractions were determined, and the inhibitory ratios (IP) were calculated according to Wu et al. [22]. The concentrations of protein were measured by the UV method using a wavelength of 280 nm, and the protein recovery rate was calculated as follows:

(1) $$R=\frac{m}{M}\times 100\text{\%}$$

R—Recovery rate of protein, %;
m—Protein content of the sample eluted from IMAC (IMAC fractions), mg;
M—Protein content of the sample before treatment of IMAC chromatographic procedures, mg.

To study the effect of the pH and the concentration of NaCl on the behavior of LFPH-I on the affinity column, the buffers used for the peptide adsorption experiment were 0.02 M phosphate buffer with different pH value (pH = 6.0–8.4) and 0.02 M phosphate buffer containing different concentrations of NaCl, respectively. The fractions were eluted with 0.02 M PBS (pH 4.0, 0.5 M NaCl).

Considering the effect of the salt on the behavior of LFPH-I on the affinity column, elution buffers with different salts and salt concentrations were used for the peptide desorption experiment.

### 3.5. Determination of Amino Acid (AA) Composition

AA composition was determined following release by acid hydrolysis as described by Li et al. [26]. Separation of free AA was performed by Agilent HPLC with an Agilent Eclipse AAA (4.6 × 150 mm, 5 μm) analytical column (Agilent Tech, Santa Clara, CA, USA).

### 3.6. Purification of ACE-Inhibitory Peptides by HPLC

The fraction collected from the IMAC-Ni^2+^ with the highest ACE inhibitory activity was collected, lyophilized, and then purified by HPLC. Elution peaks were monitored at 220 nm. Solvent A was 0.1% (*v*/*v*) trifluoroacetic acid (TFA) in water, and solvent B was 0.1% (*v*/*v*) TFA in acetonitrile. Separations were performed on Zorbax SB C18 (4.6 mm × 150 mm, 5 μm, Agilent, Santa Clara, CA, USA) and eluted with the gradient (0–20 min, 0%–50% of solvent B) at a flow rate of 1.0 mL·min^−1^. The fraction exhibiting the highest ACE inhibitory activity was collected, lyophilized, and used to identify the amino acid sequence.

### 3.7. Amino Acid Sequence Analysis

The amino acid sequence was identified using 4800 Plus MALDI TOF/TOF^TM^ Analyzer (Applied Biosystems, Redwood City, CA, USA). It was performed by School of Life Science, Guangxi University, in Nanning, China.

## 4. Conclusions

In the current study, an ACE-inhibitory peptide with the amino acid sequence Arg-Tyr-Arg-Pro (RYRP) was purified by IMAC and RP-HPLC from LFPH-I (product of hydrolysate pass through 5 kDa ultrafiltration membranes), and showed strong ACE-inhibitory activity (IC_50_ of 52 μM). This ACE-inhibitory peptide is composed of four amino acid residues and possesses a hydrophobic residue, with proline as the *C*-terminal residue. The peptide fractions eluted from the Ni^2+^ column were enriched in Met, His, Tyr, Pro, Ile, and Leu compared to the crude peptide LFPH-I.

It was found that the IMAC method can reduce two steps of separation (compared with the use of Sephadex G-15, described by Wu et al. [23]). Furthermore, there are several advantages in using IMAC, including efficiency, high purity, and low cost. Due to these advantages, IMAC would be a new technology for the separation of ACE-inhibitor.

## Figures and Tables

**Table 1:** Amino acid levels (mol %) of LFPH-I and F2eluted from IMAC.

Amino Acid	Crude Protein (LFPH-I)/mol %	F2/mol %	The Increased Percentage of Amino Acid Levels/%
ASP	9.29	9.36	0.75
Thr	5.09	5.09	0.00
Ser	5.07	5.02	−0.99
Glu	13.71	13.42	−2.12
Gly	8.09	8.21	1.48
Ala	8.86	8.96	1.13
Cys	0.39	0.07	−82.05
Val	4.89	2.87	−41.31
Met	1.49	2.40	61.07
Ile	4.33	4.62	6.70
Leu	8.56	8.97	4.79
Tyr	2.36	2.76	16.95
Phe	3.08	2.40	−22.08
Lys	8.07	7.80	−3.35
His	1.70	2.61	53.53
Arg	4.49	4.32	−3.79
Pro	7.36	7.96	8.15
Trp	3.19	3.16	−0.94

**Table 2:** The inhibitory activity of fractions separated by RP-HPLC.

Fraction	IP%
N1	51.28
N2	24.09
N3	28.96
N4	36.11
N5	73.05
N6	26.42
N7	0.00
